# Upregulation of HOTAIRM1 increases migration and invasion by glioblastoma cells

**DOI:** 10.18632/aging.202263

**Published:** 2020-12-11

**Authors:** Peng Xie, Xiang Li, Rui Chen, Yue Liu, DaChao Liu, Wenguang Liu, Gang Cui, Jinjing Xu

**Affiliations:** 1 Department of Neurosurgery, The Affiliated Huai’an Hospital of Xuzhou Medical University, The Second People’s Hospital of Huai'an, Huai’an, Jiangsu Province, China; 2 Department of Oncology, Huaian Hospital of Huaian District, Huai’an, Jiangsu Province, China; 3 Department of Central Laboratory, The Affiliated Huai’an No.1 People's Hospital of Nanjing Medical University, Huai’an, Jiangsu Province, China; 4 Department of Neurology, The Affiliated Huai’an Hospital of Xuzhou Medical University, The Second People’s Hospital of Huai'an, Huai’an, Jiangsu Province, China; 5 Department of Intensive Care Unit, The Affiliated Huai’an Hospital of Xuzhou Medical University, The Second People’s Hospital of Huai'an, Huai’an, Jiangsu Province, China; 6 Department of Image, The Affiliated Huai’an Hospital of Xuzhou Medical University, The Second People’s Hospital of Huai'an, Huai’an, Jiangsu Province, China; 7 Department of Neurosurgery, First Affiliated Hospital of Soochow University, Suzhou, China; 8 Galactophore Department, Jiangsu Huai’an Maternity and Children Hospital, Huai'an, Jiangsu Province, China

**Keywords:** HOTAIRM1, miR-153-5p, SNAI2, glioma

## Abstract

Long noncoding RNAs (lncRNAs) promote invasion and migration by glioblastoma (GBM) cells. In this study, quantitative real-time polymerase chain reaction was used to detect expression levels of the lncRNA HOTAIRM1 in GBM tissue samples and cells. The function of HOTAIRM1 was examined using wound healing assays, transwell assays, and *in vivo* experiments after GBM cells were transfected with either sh-ctrl or sh-HOTAIRM1. Luciferase reporter assays and RIP assays were performed to determine the interactions between HOTAIRM1 and miR-153-5p and between miR-153-5p and SNAI2. We also used luciferase reporter assays and ChIP assays to assess the transcriptional regulation of HOTAIRM1 by SNAI2 and CDH1. HOTAIRM1 was significantly overexpressed in GBM tissues and cells. HOTAIRM1 knockdown significantly weakened the migration and invasion by GBM cells. HOTAIRM1 was found to sponge miR-153-5p, and SNAI2 is a direct target of miR-153-5p. In addition, SNAI2 was shown to force HOTAIRM1 expression through directly promoting transcription and suppressing the negative regulation of CDH1 on transcription. Our results indicate a positive feedback loop between HOTAIRM1 and SNAI2, and suggest that the lncRNA HOTAIRM1 is a potential biomarker and therapeutic target in GBM.

## INTRODUCTION

Glioma accounts for more than half of the primary tumors in the central nervous system of adults. Glioblastoma multiforme (GBM) is the most malignant subtype of glioma [1, 2]. Despite aggressive therapy, including resection, chemotherapy, and radiotherapy, GBM patients still have a poor prognosis [2]. Thus, studying the biology, genetics, and epigenetic alterations in GBM, especially the mechanisms underlying the invasive phenotype, is vital to improve the prognosis of patients with GBM.

Epithelial-to-mesenchymal transition (EMT) is the shift of cells from the epithelial to mesenchymal phenotype, which results in increased invasiveness of GBM cells [3, 4]. Various factors control the EMT process in GBM [5, 6]. Long noncoding RNAs (lncRNAs) are a type of RNA composed of more than 200 nucleotides [7]. Recent studies indicate that lncRNAs affect a variety of biological processes, including EMT [7–9]. One role of lncRNAs is to act as competitive endogenous RNAs (ceRNAs), which means that lncRNAs inhibit the expression of specific mRNAs by sponging microRNAs (miRNAs) [10, 11]. However, more information about how lncRNAs control the expression of EMT-associated genes in GBM cells is needed.

In this study, we provide evidence that the lncRNA HOTAIRM1 contributes to GBM migration and invasion *in vitro* and *in vivo*. We further show that HOTAIRM1 upregulates SNAI2 by sponging miR-153-5p and induces EMT. In turn, upregulated SNAI2 increases HOTAIRM1. Our results indicate that HOTAIRM1 may be a therapeutic target for GBM.

## RESULTS

### HOTAIRM1 is upregulated in GBM tissues and cells

**Figure 1:**
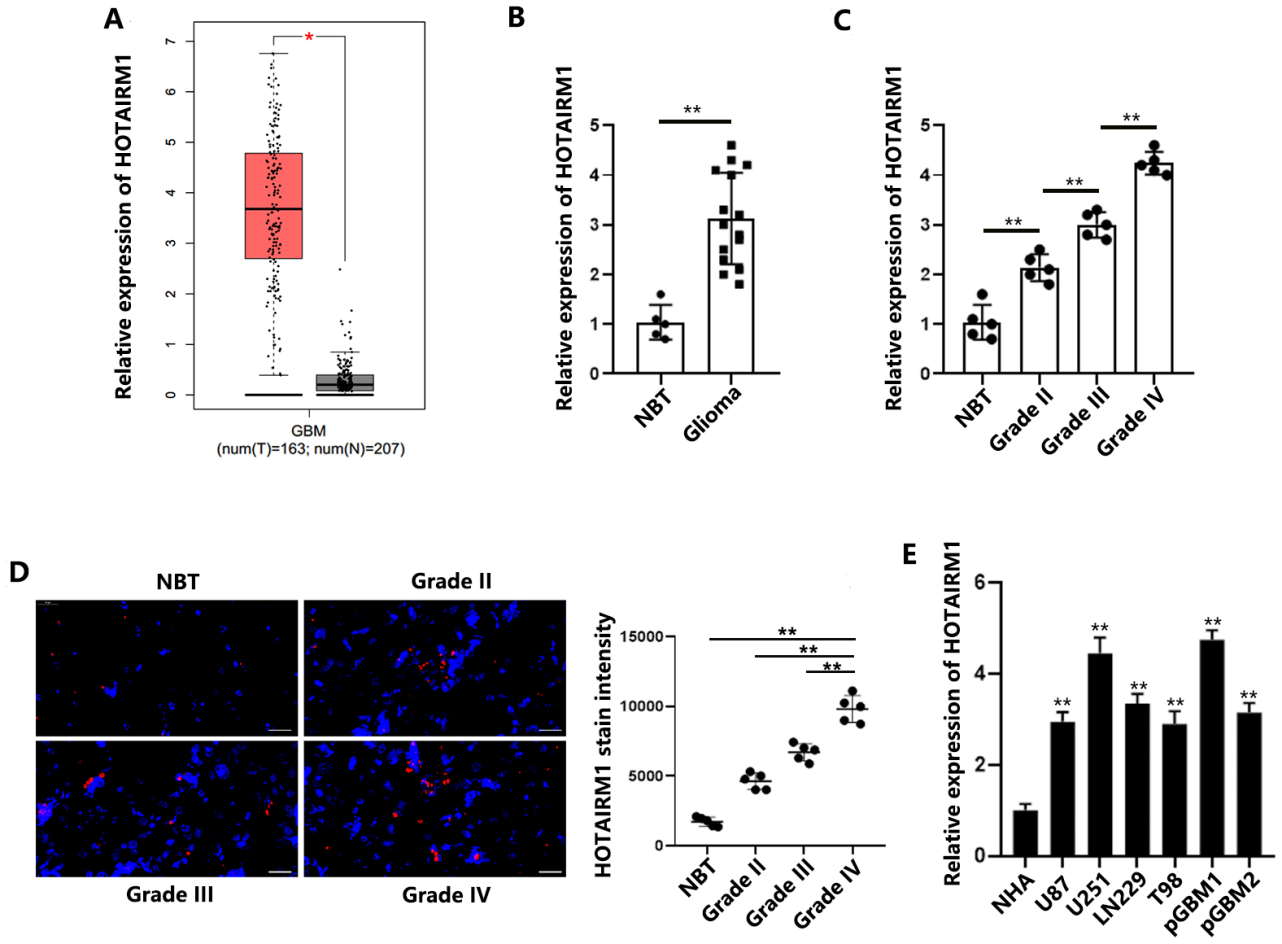
**HOTAIRM1 is upregulated in GBM tissues and cells.** (**A**) HOTAIRM1 was significantly upregulated in GBM samples (n = 163) compared with corresponding normal tissues (n = 207) according to the GEPIA database. (**B**) Relative HOTAIRM1 expression was analyzed in 5 normal brain tissues and 15 glioma tissues (5 grade II, 5 grade III, and 5 grade IV). (**C**) Relative expression of HOTAIRM1 in normal brain tissues and different grades of glioma tissues was determined. (**D**) Representative images of FISH analysis of HOTAIRM1 expression in normal brain tissues and different grades of glioma tissues. Scale bar = 50 μm. (**E**) Relative expression of HOTAIRM1 in NHAs and 6 GBM cells was determined. *P < 0.05, **P < 0.01.

To define the role of HOTAIRM1 in GBM, we examined the expression levels of HOTAIRM1 using the GEPIA data set (http://gepia.cancer-pku.cn/). The box plots show that HOTAIRM1 expression is significantly higher in GBM tissues than normal brain tissues (Figure 1A). Similarly, we quantified HOTAIRM1 levels in five normal brain tissue samples and 15 glioma samples that included three World Health Organization (WHO) glioma grades. The results showed that HOTAIRM1 was significantly increased in glioma tissues compared with normal brain tissues (Figure 1B). Furthermore, HOTAIRM1 levels gradually increased with increasing WHO grade, and grade IV gliomas (GBMs) had the highest level of HOTAIRM1 (Figure 1C). Fluorescence in situ hybridization (FISH) analysis confirmed the qRT-PCR results (Figure 1D). Expression of HOTAIRM1 was also determined in a panel of GBM cell lines (U87, U251, LN229, T98, pGBM1, and pGBM2) and normal human astrocyte cells. We found that HOTAIRM1 expression was upregulated in the GBM cell lines compared with the normal human astrocyte cells (Figure 1E). To summarize, HOTAIRM1 level was positively associated with glioma tumor grade, and GBM had the highest expression level.

### HOTAIRM1 promotes migration and invasion of GBM cells *in vitro* and tumor growth *in vivo*

**Figure 2:**
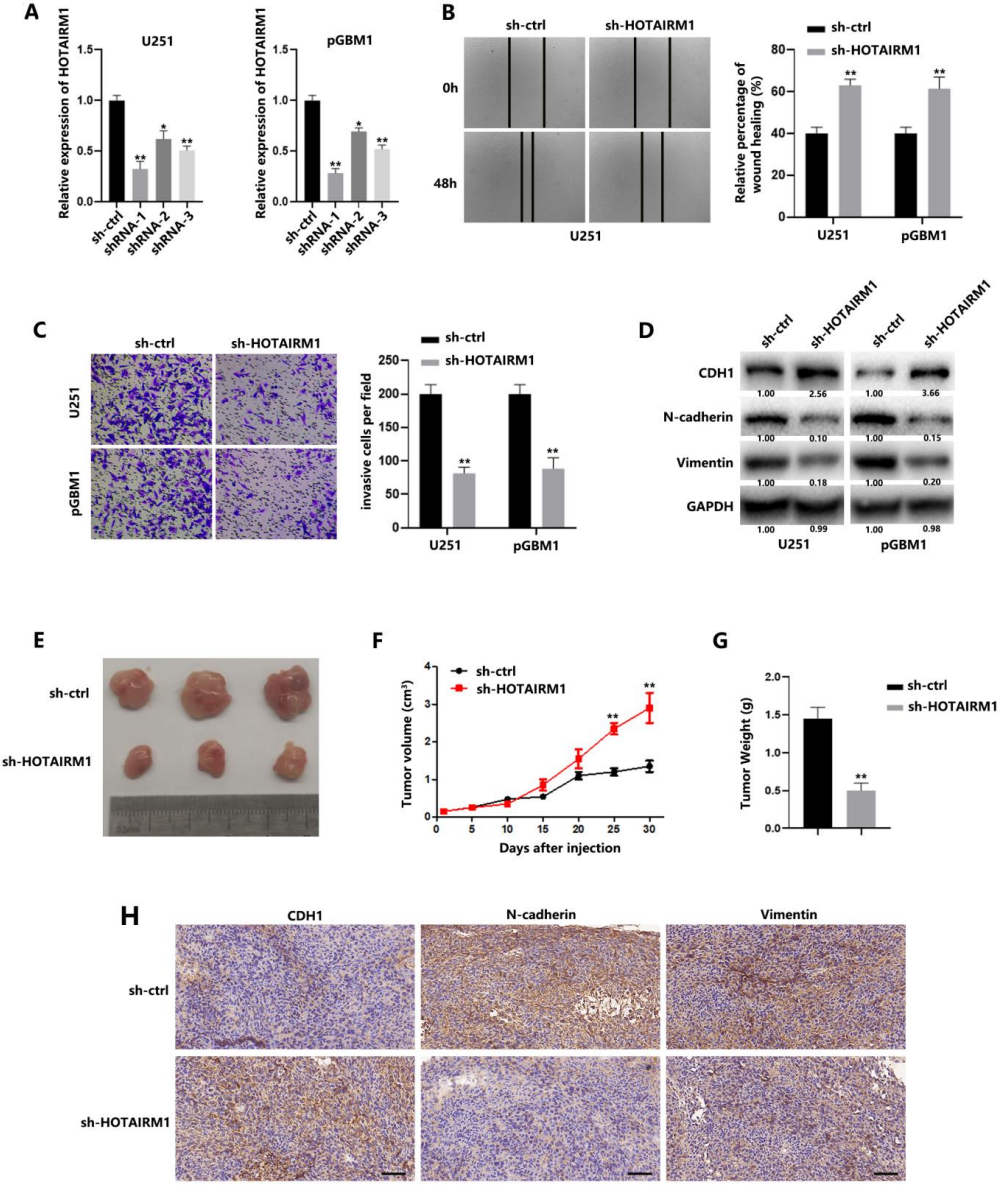
**HOTAIRM1 promotes migration and invasion of GBM cells and tumor growth *in vivo*.** (**A**) Relative expression of HOTAIRM1 in cells transfected with HOTAIRM1 shRNA and negative control. (**B**) Wound healing assays were used to analyze migration of GBM cells. (**C**) Matrigel invasion assays were used to analyze invasion of GBM cells. (**D**) EMT-associated proteins in GBM cells were determined using western blotting. (**E**) Representative images of subcutaneous tumors originated from sh-ctrl– or sh-HOTAIRM1–transfected pGBM1 cells on the da\ ys indicated. (**F**) Growth curve of tumors originated from sh-ctrl– or sh-HOTAIRM1–transfected pGBM1 cells. (**G**) Weight of tumors originated from sh-ctrl– or sh-HOTAIRM1–transfected pGBM1 cells. (**H**) Representative IHC results of CDH1, N-cadherin and Vimentin in tumors. *P < 0.05, **P < 0.01.

To identify the functional relevance of dysregulated HOTAIRM1, U251 and pGBM1 cells, which express higher levels of HOTAIRM1 than other GBM cell lines, were selected for further analysis. Lentivirus stably expressing short hairpin RNAs targeting HOTAIRM1 (shRNA-1, shRNA-2 and shRNA-3), as well as the scramble sequence (sh-ctrl), were introduced into GBM cells, and qRT-PCR analysis confirmed the knockdown of HOTAIRM1 in U251 and pGBM1 cells (Figure 2A). Wound healing and Matrigel transwell assay results demonstrated that knockdown of HOTAIRM1 significantly weakened the migration and invasion of GBM cells (Figure 2B, 2C). CDH1 was upregulated and N-cadherin and vimentin were downregulated in GBM cells transfected with sh-HOTAIRM1, which indicates that HOTAIRM1 knockdown suppresses EMT in GBM cells (Figure 2D). These results were confirmed by sh-HOTAIRM1-2 transfection (Supplementary Figure 1A–1C).

To determine the effect of HOTAIRM1 on tumor growth, we subcutaneously injected pGBM1 cells pretransfected with sh-HOTAIRM1 into nude mice. Not surprisingly, tumor progression from HOTAIRM1-downregulated pGBM1 cells was much slower than that of a control tumor raised from control cells (Figures 2E, 2F, 2G). IHC results indicated that decreased HOTAIRM1 contributes to EMT process in GBM (Figure 2H).

### HOTAIRM1 serves as a molecular sponge for miR-153-5p

**Figure 3:**
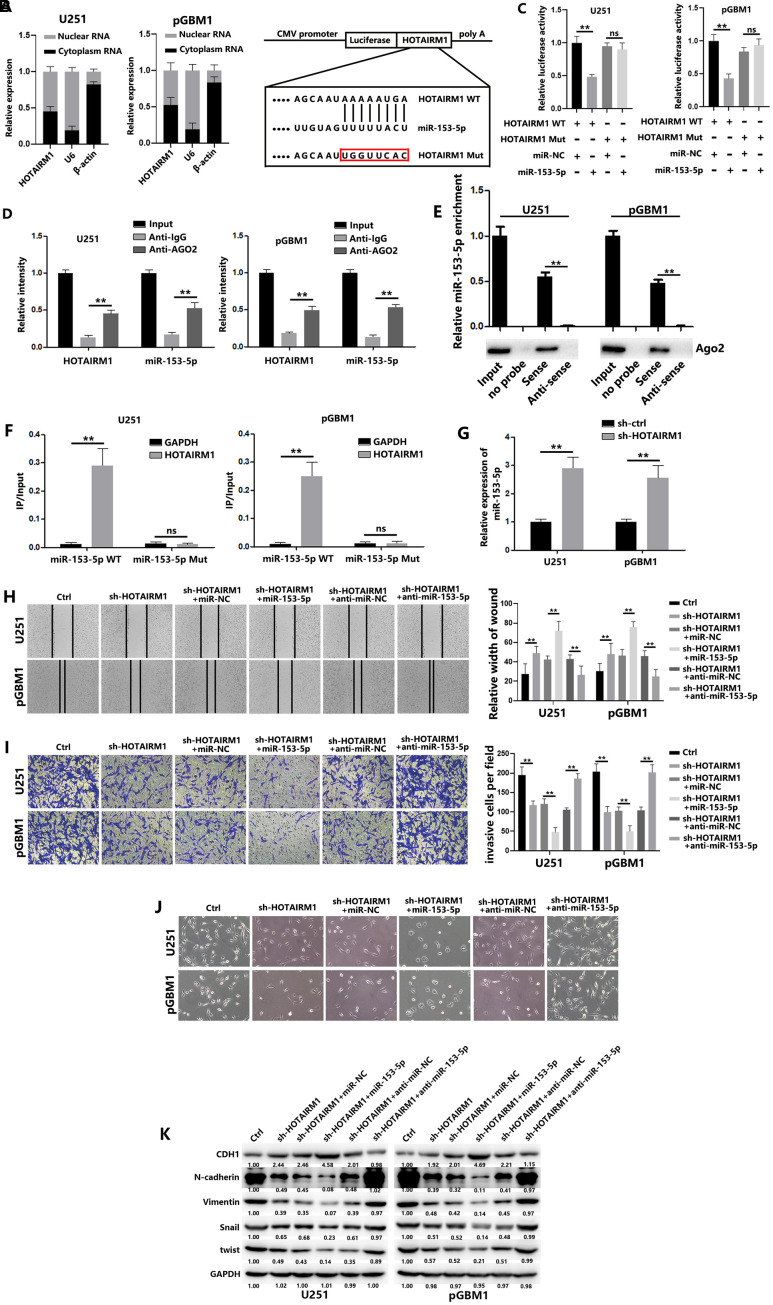
**HOTAIRM1 serves as a molecular sponge for miR-153-5p.** (**A**) HOTAIRM1 expression in the nucleus and cytoplasm of GBM cells was measured using qRT-PCR. U6 (nuclear retained) and β-actin (exported to cytoplasm) were used as controls. (**B**) Schematic diagram shows the putative miR-153-5p binding sites with HOTAIRM1. The sequences of wild-type HOTAIRM1 and mutant HOTAIRM1 are listed as well. (**C**) Luciferase reporter gene assays were performed to measure the luciferase activity in GBM cells. (**D**) RNA immunoprecipitation (RIP) assays were performed to determine HOTAIRM1 and miR-153-5p RNA enrichment in immunoprecipitated (IP) complex. Anti-immunoglobulin G (IgG) was used as the control. (**E**) Relative expression of miR-153-5p and the level of Ago2 in the products of HOTAIRM1 based pull-down assays. (**F**) The biotinylated miR-153-5p WT or miR-153-5p Mut was transfected into GBM cells. qRT-PCR was performed to quantify the RNA levels of HOTAIRM1 and GAPDH. Relative ratios of the input of IP were analyzed. (**G**) Relative expression of miR-153-5p in GBM cells was analyzed after transfection with sh-ctrl or sh-HOTAIRM1. (**H**) Wound healing assays were used to analyze migration of GBM cells. (**I**) Matrigel invasion assays were used to analyze invasion of GBM cells. (**J**) Morphological changes of GBM cells were imaged to analyze EMT process of GBM cells. (**K**) EMT-associated proteins in GBM cells were determined using western blotting. *P < 0.05, **P < 0.01.

To investigate the mechanisms of HOTAIRM1, we analyzed the subcellular site of HOTAIRM1 in GBM cells. We isolated cytoplasmic and nuclear RNAs and performed qRT-PCR analysis. The results indicated that HOTAIRM1 is located in both the cytoplasm and nucleus (Figure 3A). Using the Diana Tools bioinformatic website (http://diana.imis.athena-innovation.gr/DianaTools/index.php), we found a reciprocal sequence to HOTAIRM1 in miR-153-5p. To confirm the association, we constructed HOTAIRM1 wild-type (WT) and HOTAIRM1 mutant (Mut) luciferase reporter genes (Figure 3B). Then, we co-transfected the luciferase reporter genes with miR-153-5p or miR-NC. Overexpression of miR-153-5p significantly weakened HOTAIRM1 WT luciferase activity compared with the control group. However, overexpression of miR-153-5p did not affect the luciferase activity of mutant HOTAIRM1 (Figure 3C). Next, we performed anti-AGO2–based RIP assays and found that HOTAIRM1 and miR-153-5p were concentrated preferentially in the extraction of GBM cells (Figure 3D). These results demonstrated the connection between HOTAIRM1 and miR-153-5p via AGO2 in GBM cells. In addition, RNA pull-down assays demonstrated that HOTAIRM1 was more enriched in the pull-down product of wild-type miR-153-5p than in the mutant-type miR-153-5p (Figure 3E). qRT-PCR results indicated that the miR-153-5p level was significantly increased after downregulation of HOTAIRM1 (Figure 3F).

To explore whether miR-153-5p affects HOTAIRM1-mediated invasive behavior, we co-transfected miR-153-5p mimics or anti-miR-153-5p together with sh-HOTAIRM1 and then studied the migration and invasion of GBM cells. We found that miR-153-5p overexpression increased the suppressive effect of sh-HOTAIRM1 transfection on migration and invasion. Meanwhile, miR-153-5p inhibition reversed the effect of HOTAIRM1 knockdown on migration and invasion in GBM cells (Figures 3H and 3I). We also analyzed EMT-associated protein markers. The results suggest that miR-153-5p overexpression suppresses the EMT process. In addition, downregulation of miR-153-5p may counteract the negative effect on EMT generated by HOTAIRM1 decrease (Figure 3J, 3K). Together, these data suggest that HOTAIRM1 promotes GBM progression by sponging miR-153-5p.

### SNAI2 is a direct target of miR-153-5p

**Figure 4:**
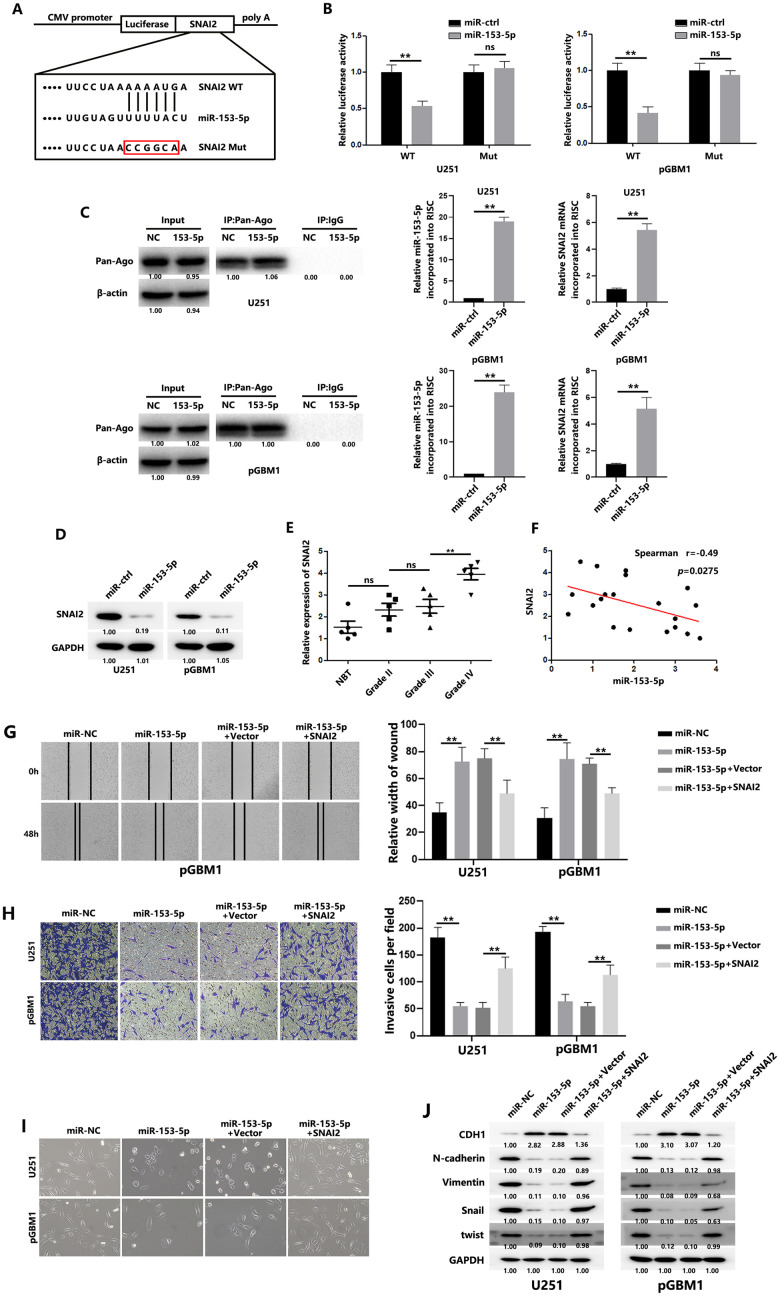
**SNAI2 is a direct target of miR-153-5p.** (**A**) Schematic diagram shows the predicted miR-153-5p binding sites with the 3′-UTR of SNAI2. The sequences of wild-type and mutant 3′-UTR of SNAI2 are also listed. (**B**) Luciferase assays were performed to measure the luciferase activity in GBM cells. (**C**) (Left): Immunoprecipitation of the Ago2/RISC using the Pan-Ago2 antibody in U251 or pGBM1 cells overexpressing miR-NC or miR-153-5p. IgG was used as a negative control, and β-actin was used as an internal control. (Middle): qRT-PCR analysis of miR-153-5p incorporated into RISC in U251 or pGBM1 cells overexpressing miR-153-5p compared to the levels in the control. U6 RNA was used as an internal control. (Right): qRT-PCR of SNAI2 incorporated into RISC in U251 or pGBM1 cells overexpressing miR-153-5p. GAPDH RNA was used as an internal control. (**D**) Western blot analysis indicated that SNAI2 expression levels were decreased in cells with miR-153-5p overexpression. (**E**) SNAI2 expression levels in 5 normal brain tissues and 15 glioma specimens (5 glioma tissues in each group: WHO grades II, III, and IV) were examined using qRT-PCR. (**F**) Spearman correlation analysis was used to confirm the correlation between the SNAI2 and miR-153-5p levels in 20 human glioma specimens. (**G**) Wound healing assays were used to analyze migration of GBM cells. (**H**) Matrigel invasion assays were used to analyze invasion of GBM cells. (**I**) Morphological changes of GBM cells were imaged to analyze EMT process of GBM cells. (**J**) EMT-associated proteins in GBM cells were determined by western blotting. *P < 0.05, **P < 0.01.

Using the TargetScan bioinformatic website (http://www.targetscan.org/vert_72/), we found a putative binding site of miR-153-5p in the SNAI2 3′- UTR (Figure 4A). Luciferase reporter assays were performed to determine whether miR-153-5p directly binds to the predicted site in SNAI2. MiR-153-5p overexpression was significantly decreased in the wild-type reporter plasmid of SNAI2 compared with the mutated reporter plasmid of SNAI2 (Figure 4B). We also performed RNA-ChIP analysis to confirm the interaction between miR-153-5p and SNAI2. Enrichment of SNAI2 in the Ago2/RNA-induced silencing complex (RISC) was elevated in miR-153-5p–overexpressing cells (Figure 4C, right). Transiently overexpressing miR-153-5p in GBM cells reduced SNAI2 expression level (Figure 4D). In addition, SNAI2 expression was significantly greater in the 15 gliomas than in the five normal brain tissues (Figure 4E), and we observed an inverse correlation between SNAI2 and miR-153-5p levels (Figure 4F). As expected, SNAI2 plasmid transfection reversed the suppressive effect of miR-153-5p on the migration and invasion of GBM cells and the EMT process (Figures 4G–4J), while si-SNAI2 reversed the supportive effect of anti-miR-153-5p on EMT process of GBM cells (Supplementary Figures 2A–2D). Above all, miR-153-5p was found to directly target SNAI2.

### SNAI2 positively regulates HOTAIRM1 in GBM cells

**Figure 5:**
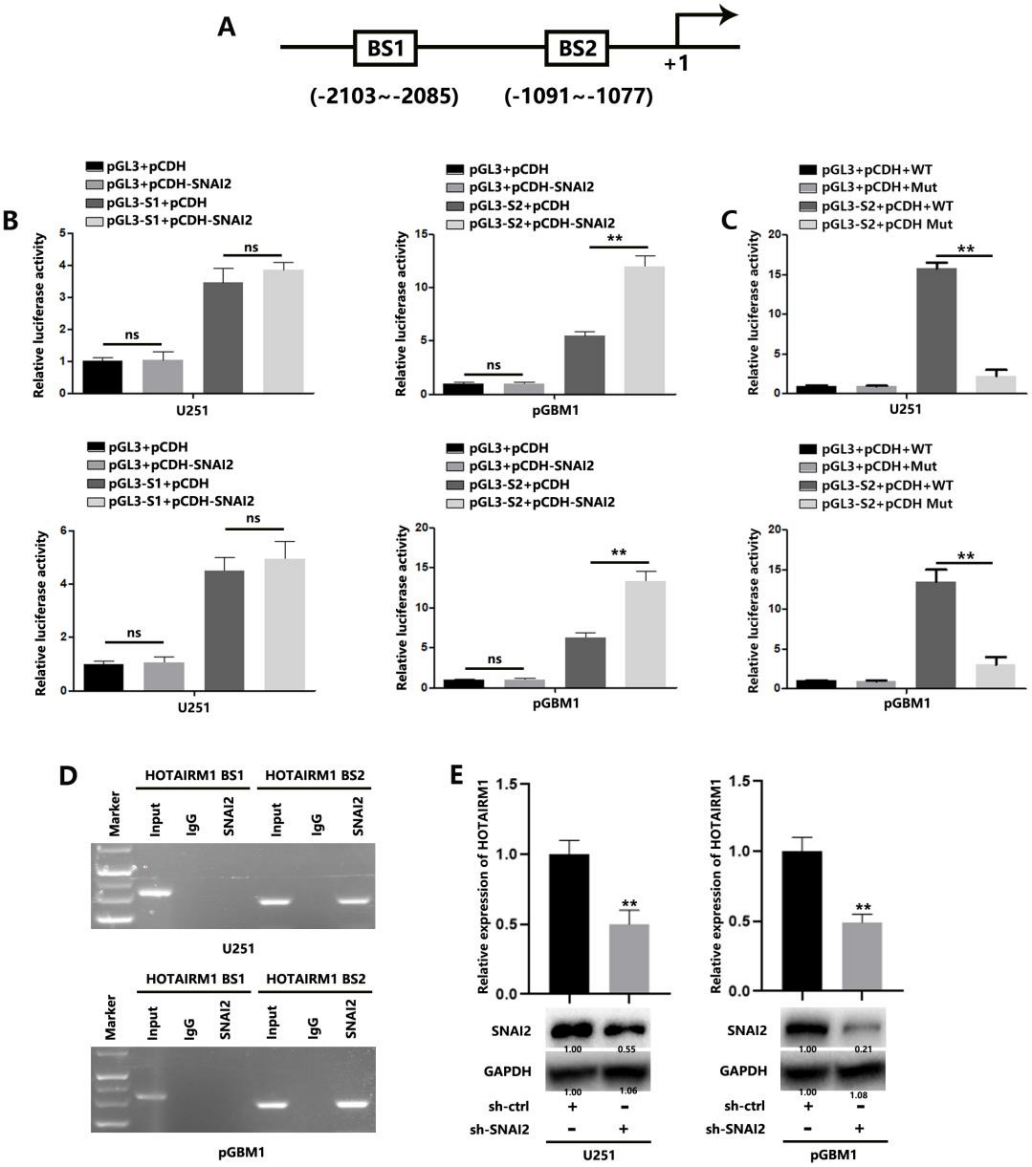
**SNAI2 transcriptionally regulates HOTAIRM1 in GBM cells.** (**A**) Two potential SNAI2-binding sites in the HOTAIRM1 promoter region were predicted using the high-quality transcription factor binding profile database (JASPAR). (**B**) The two predicted regions were transcriptionally responsive to SNAI2 overexpression, as shown in luciferase reporter assays. (**C**) Wild-type or mutant luciferase reporter were transfected into GBM cells, and the luciferase reporter activity were analyzed. (**D**) SNAI2 bound to both predicted binding sites in HOTAIRM1 promoter, as shown in ChIP assays. (**E**) SNAI2 shRNA reduced the levels of HOTAIRM1 in U251 and pGBM1 cells, as shown in qRT-PCR analysis (top) and western blotting (bottom).

Results of in silico analysis suggested two possible SNAI2-binding sites (SNAI2-BS1: –2103 to –2085 bp; and SNAI2-BS2: –1091 to –1077 bp) in the HOTAIRM1 transcriptional initiation site (Figure 5A). Luciferase reporter assays indicated that the BS2 region is responsible for SNAI2-mediated HOTAIRM1 transcription (Figure 5B), while SNAI2 binding site mutation significantly inhibited the interaction between HOTAIRM1 and SNAI2 (Figure 5C). We performed ChIP analyses, which confirmed that SNAI2 interacts with the BS2 binding site, not the BS1 binding site, in the HOTAIRM1 promoter (Figure 5D). In addition, silencing SNAI2 reduced the expression of HOTAIRM1 in GBM cells (Figure 5E). These results indicated that SNAI2 could directly promote HOTAIRM1 transcription, inducing increased HOTAIRM1 in GBM cells.

**Figure 6:**
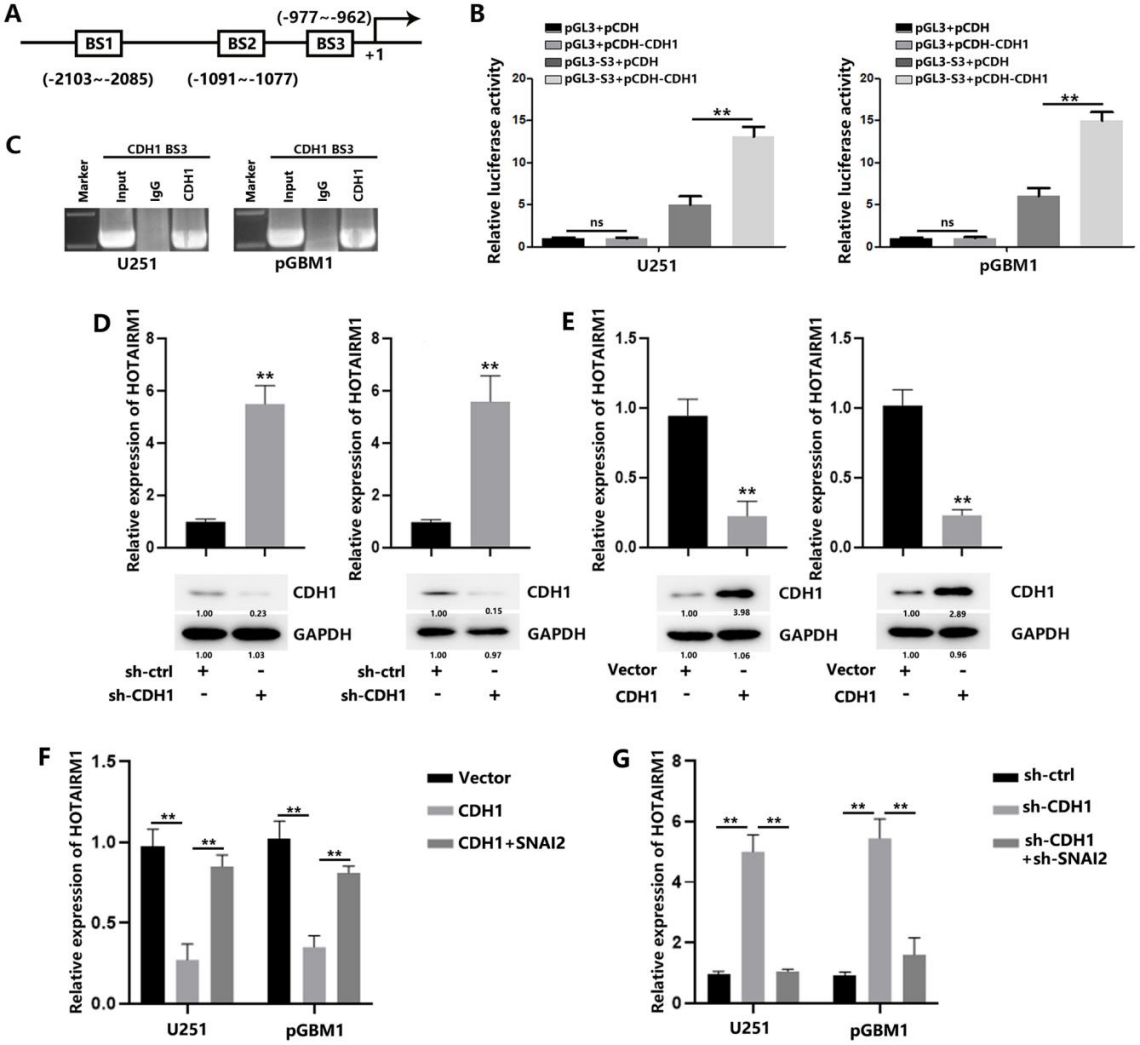
**SNAI2 suppress CDH1 to induce HOTAIRM1 in GBM cells.** (**A**) The potential CDH1-binding sites (BS3) in the HOTAIRM1 promoter region was predicted using the high-quality transcription factor binding profile database (JASPAR). (**B**) The predicted region was transcriptionally responsive to CDH1 overexpression, as shown in luciferase reporter assays. (**C**) CDH1 bound to both predicted binding sites in HOTAIRM1 promoter, as shown in ChIP assays. (**D**) Relative expression of HOTAIRM1 in GBM cells transfected with sh-ctrl or sh-CDH1. (**E**) Relative expression of HOTAIRM1 in GBM cells transfected with vector or CDH1. (**F**) Relative expression of HOTAIRM1 in GBM cells transfected with vector or CDH1 or co-transfected with CDH1 and SNAI2. (**G**) Relative expression of HOTAIRM1 in GBM cells transfected with sh-ctrl or sh-CDH1 or co-transfected with sh-CDH1 and sh-SNAI2.

**Figure 7:**
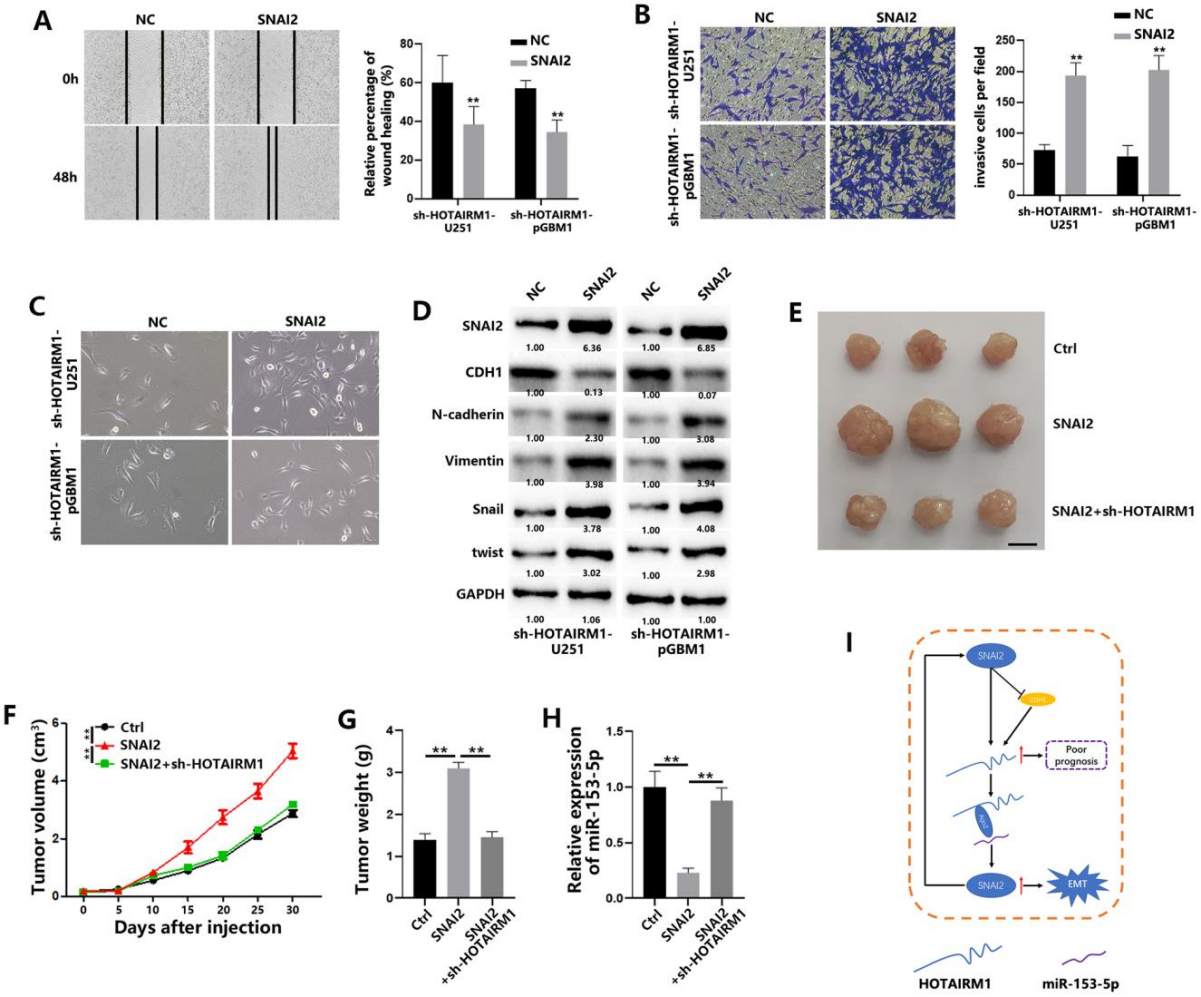
**SNAI2 promotes migration and invasion of GBM cells and tumor growth *in vivo*.** (**A**) Wound healing assays were used to analyze migration of GBM cells. (**B**) Matrigel invasion assays were used to analyze invasion of GBM cells. (**C**) Morphological changes of GBM cells were imaged to analyze EMT process of GBM cells. (**D**) EMT-associated proteins in GBM cells were determined by western blotting. (**E**) Representative images of subcutaneous tumors originated from transfected pGBM1 cells on the days indicated. (**F**) Growth curve of tumors originated from transfected pGBM1 cells. (**G**) Weight of tumors originated from transfected pGBM1 cells. (**H**) Relative expression of miR-153-5p in tumors originated from transfected pGBM1 cells. (**I**) Schematic illustration of proposed model depicting important role of the lncRNA HOTAIRM1 in regulating cell migration and invasion. *P < 0.05, **P < 0.01.

Previous results suggested that SNAI2 was proved as a repressor to suppress CDH1 expression. In this study, we found that SNAI2 could induce decreased CDH1 expression in GBM cells (Figure 7D). Therefore, we tend to figure out additional detailed mechanism explaining transcriptionally HOTAIRM1 activation. Similarly, we also found a putative binding site of CDH1, BS3, on HOTAIRM1 promoter (Figure 6A). Luciferase reporter assays showed that CDH1 overexpression induced increased luciferase activity, indicating the interaction between CDH1 and HOTAIRM1 promoter. (Figure 6B). CHIP analysis confirmed the regulation of CDH1 on HOTAIRM1 (Figure 6C). CDH1 down-regulation increased HOTAIRM1 expression levels (Figure 6D), while CDH1 overexpression decreased HOTAIRM1 expression level (Figure 6E). In addition, the decreased HOTAIRM1 levels caused by CDH1 could be rescued by SNAI2 (Figure 6F), while increased HOTAIRM1 caused by CDH1 knockdown could be rescued by SNAI2 knockdown (Figure 6G). These results indicated that CDH1 is a negative regulator of HOTAIRM1; And, SNAI2 could also promote HOTAIRM1 transcription through suppressing the negative regulation of CDH1 on HOTAIRM1.

Next, we also investigated the connection between SNAI2 and HOTAIRM1 through *in vitro* experiments. As shown in Figure 7A, 7B, SNAI2 overexpression reversed the decreased migration and invasion of GBM cells caused by HOTAIRM1 knockdown. In addition, EMT process was significantly promoted when SNAI2 was overexpressed in HOTAIRM1-knockdown cells (Figure 7C, 7D). Meanwhile, *in vivo* studies showed that SNAI2 could promote the tumor progression, and HOTAIRM1 knockdown reversed this results (Figure 7E–7G). qRT-PCR results showed that SNAI2 overexpression led to decreased miR-153-5p, and sh-HOTAIRM1 co-transfection increased the levels of miR-153-5p in tumors (Figure 7H).

Together, these data indicate that SNAI2 could increase lncRNA HOTAIRM1 in GBM cells, indicating a positive feedback loop between HOTAIRM1 and SNAI2 (Figure 7I).

## DISCUSSION

LncRNAs exert specific functions in the development of various tumors, and targeting lncRNAs is being investigated as a potential tumor treatment strategy [12–14]. Increasing evidence suggests that lncRNAs may regulate the malignant behavior of glioma and contribute to chemotherapy resistance [15, 16]. In this study, we investigated the lncRNA HOTAIRM1, which is overexpressed in GBM tissues compared with low-grade glioma and normal brain tissues. HOTAIRM1 was previously shown to regulate differentiation and cell cycle progression in myeloid cells [17, 18]. Because of its aggressive nature, GBM cannot be completely resected. Therefore, we explored the contribution of HOTAIRM1 to the invasiveness of GBM. We found that increased HOTAIRM1 expression promoted migration and invasion by increasing EMT in GBM cells. *In vivo* results confirmed that HOTAIRM1 promotes tumor progression in GBM.

Next, we investigated the fundamental mechanisms underlying the regulation of HOTAIRM1 on GBM invasion. A recent article reported that HOTAIRM1 could demethylate histone H3K9 and H3K27, reducing HOXA1 DNA methylation [19]. In addition to exploring this theory, we wanted to further elucidate the role of HOTAIRM1 in GBM cells. One important role of lncRNA is to regulate gene expression by sponging miRNAs [10, 11]. For example, the lncRNA MT1JP regulates FBXW7 as a ceRNA by interacting with miR-92a-3p in gastric cancer [20]. However, the role of HOTAIRM1 as a ceRNA in GBM is still unclear. Here, we confirmed that when HOTAIRM1 binds to miR-153-5p, the function of miR-153-5p is inhibited. Previous studies have shown that miR-153 acts as a tumor suppressor, inhibiting the emergence and progression of cancer [21–23]. Consistent with these results, our study confirms the oncogenic function of HOTAIRM1 and that miR-153-5p can reverse the pro-invasive effects of HOTAIRM1. Finally, we were able to clarify the relationship between HOTAIRM1 and SNAI2, which acts as a transcriptional promoter and is involved in EMT [8, 24]. We found that HOTAIRM1 expression is determined by transcriptional regulation of SNAI2. Interestingly, we also found that SNAI2 was regulated by miR-153-5p, as shown in Figure 4. Thus, we found a feedback loop whereby HOTAIRM1 induces SNAI2 through posttranscriptional regulation of miR-153-5p and, in turn, SNAI2 controls HOTAIRM1 expression.

In summary (Figure 7I), we demonstrated that upregulated HOTAIRM1 promotes the migration and invasion of GBM cells. HOTAIRM1 induces SNAI2 by sponging miR-153-5p, and SNAI2 in turn transcriptionally increases HOTAIRM1 expression. Meanwhile, SNAI2 could decrease CDH1 expression, inhibiting the negative regulation of CDH1 on HOTAIRM1 transcription. Togehter, targeting HOTAIRM1 is a possible therapeutic strategy for GBM.

## MATERIALS AND METHODS

### Cell culture

The GBM cell lines U87, U251, LN229, and T98 were purchased from the Chinese Academy of Sciences Cell Bank (Shanghai, China) and were maintained in Dulbecco’s modified Eagle’s medium (DMEM) supplemented with 10% fetal bovine serum (FBS), 100 units of penicillin/mL, and 100 ng of streptomycin/mL. Two primary human GBM cells (pGBM1 and pGBM2) were obtained from primary human GBM samples as described previously [25]. For cell culture, all cells were incubated in 5% CO_2_ at 37° C.

### Clinical specimens

Fifteen glioma tissues and five paratumor samples were collected from GBM patients at the Department of Neurosurgery, the Affiliated Huai'an Hospital of Xuzhou Medical University, the Second People’s Hospital of Huai'an. The sample tissues were obtained during surgery and immediately frozen for storage. The experimental protocol was approved by the Ethics Committee of Xuzhou Medical University. All patients signed the informed consent.

### RNA extraction and quantitative reverse transcription polymerase chain reaction (qRT-PCR) assay

Extraction of total RNA of clinical samples and cultured cells was performed using Trizol (Invitrogen) according to the manufacturer’s protocol. qRT-PCR analyses were performed using Gotaq Green Master Mix (TaKara, Nanjing, China). Fold change was determined as 2^-ΔΔCt^ in gene expression. The primers used in this study were available in Supplementary Table 1.

### Western blot analysis

Western blot analyses were performed in line with previously described protocols [26]. Antibodies against CDH1 and N-cadherin (Cell Signaling Technology, USA), vimentin (Abcam, USA), and GAPDH and β-actin (Santa Cruz Biotechnology, USA) were used for Western blot analysis. The information of antibodies used in this study were available in Supplementary Table 1

### Plasmid construction and transfection

For reliable transfection, vector-based short hairpin RNAs (shRNAs) against HOTAIRM1 sequences and scrambled sequences were constructed. U251 and pGBM1 cells were used to establish stable cell lines and treated with puromycin at 48 hours after infection. For short-term transfection, miR-153-5p mimics and miR-153-5p inhibitor were purchased from Genechem (Shanghai, China) and transfected into cells using a previously described method [27]. The information of antibodies used in this study were available in Supplementary Materials.

### Fluorescence in situ hybridization (FISH) analysis

HOTAIRM1 expression in normal brain tissue and glioma samples was determined using FISH, as described previously [28]. The probe sequence (5’-3’) were shown as follows. HOTAIRM1-1: TGCGCGCGCCCGACTCCGCTGCCCG HOTAIRM1-2: TTACTCATTCCTGGAGTTGGGGGTTTCTGTAGGCA.

### Cell migration and invasion assay

To evaluate cell migration ability, wound healing assays were performed. Briefly, GBM cells were seeded to form the cell monolayer. Then, we used a sterile 20-μL pipette tip to shape the wounds. Cells were washed with phosphate-buffered saline twice and sustained in FBS-starved medium to remove the interference of cell proliferation. Then, cells were observed at indicated times.

To evaluate cell invasiveness, transwell invasion assays were performed. Briefly, Matrigel was introduced into transwell inserts for 30 minutes. Then, GBM cells were seeded onto the upper flat of transwell inserts. Forty-eight hours later, we removed the cells remaining on the upper surface and used 0.1% crystal violet to stain the cells at the bottom of the wells.

### RNA isolation of nuclear and cytoplasmic fractions

The Nuclear/Cytoplasmic Isolation Kit (Biovision) was used to isolate nuclear and cytoplasmic RNA separately. HOTAIRM1, U6, and β-actin expression was analyzed using qRT-PCR.

### Luciferase reporter assay

To confirm the connection between HOTAIRM1 and miR-153-5p, we constructed the wild-type reporter vector (pmirGLO-HOTAIRM1-wt) and the mutant reporter vector (pmirGLO-HOTAIRM1-Mut) by cloning wild-type or mutant HOTAIRM1 cDNA into the pmirGLO Dual-Luciferase miRNA Target Expression Vector (Promega). GBM cells were co-transfected with miR-153-5p or miR-NC and reporter vectors. To verify whether miR-153-5p could bind on 3′-UTR fragments of SNAI2, we cloned the wild-type or mutant fragments of SNAI2 3′-UTR into the pmirGLO vector. Meanwhile, we co-transfected miR-NC or miR-153-5p mimics together with SNAI2-WT or SNAI2-Mut vehicle into GBM cells using Lipofectamine 2000 (Invitrogen). Twenty-four hours after transfection, we administered a Dual-Luciferase Reporter Assay kit (Promega) to assess the luciferase activity.

To confirm the regulation of SNAI2 on HOTAIRM1, cells were transfected with the pGL3-based constructs containing HOTAIRM1 promoter together with *Renilla* luciferase plasmids. Twenty-four hours later, firefly and *Renilla* luciferase activity was examined by the Dual-Luciferase Reporter Assay System (Promega), and *Renilla* activity was used to normalize firefly activity.

### RNA immunoprecipitation (RIP)

The Imprint RNA Immunoprecipitation Kit (Sigma-Aldrich) was used for the RIP assay. U251 and pGBM1 cells were washed and fixed. Then, cells were centrifugated with 1500 × g for 15 minutes at 4° C. The cell sedimentation was collected and resuspended using lysis buffer. RNA was immunoprecipitated with antibody against Ago2 or antibody against IgG as control. After incubation for 6 hours at 4° C, the RNA was purified after removing protein beads. Then, qRT-PCR analysis was performed.

### RNA pull-down assay

To identify the direct interaction between HOTAIRM1 and miR-153-5p, miR-153-5p–based pull-down assays were performed, as previously described [29]. Briefly, wild-type or mutant miR-153-5p (“UUUUUACU” was mutated to “CCAAAGCA”) was labeled with biotin at the 3′-end and was transfected into U251 and pGBM1 cells. Then, cells were lysed and incubated with streptavidin-coated magnetic beads (Life Technologies). The biotin-coupled product was pulled down, and the enrichment of HOTAIRM1 and Ago2 in product was analyzed by qRT-PCR and western blot.

In addition, HOTAIRM1-based pull-down assays were performed. Similarly, wild-type or mutant HOTAIRM1 (“UGGUUCAC” was mutated to “UCCUAACA”) was labeled with biotin at the 3′-end and was transfected into U251 and pGBM1 cells. Then, cells were lysed and incubated with streptavidin-coated magnetic beads (Life Technologies). The biotin-coupled product was pulled down, and the enrichment of Ago2 and miR-153-5p in product was analyzed by qRT-PCR and western blot.

### Chromatin immunoprecipitation (ChIP)

For the ChIP assay, the Pierce Magnetic ChIP Kit (Thermo Scientific) was used according to the manufacturer’s protocol. Briefly, U251 and pGBM1 cells were washed and fixed. To generate DNA-protein complex, cells were incubated for 10 minutes. To generate the chromatin fragments, cell lysates were sonicated and immunoprecipitated with SNAI2 antibody or IgG antibody as a negative control. Next, coupled DNA fragments underwent qPCR.

### Subcutaneous xenograft studies

All experimental mice were purchased from the Model Animal Research Center of Nanjing University. To further study the role of HOTAIRM1 in GBM, 1 × 10^7^ GBM cells were injected into 6-week-old male BALB/c nude mice. Tumor growth was determined by length (L) and width (W), which were measured at certain time after the injection. The formula, V = (L×W^2^) × 0.5, was used to calculate tumor volume (V).

### Statistic analysis

GraphPad Prism 8.0 software was used to perform statistical analysis. All data come from three independent experiments and represent the mean ± standard deviation. Student’s *t* test was conducted to compare the difference between paired groups. *P* < 0.05 was considered statistically significant.

### Data availability statement

The data that support the findings of this study are available from the corresponding author upon request

## Supplementary Material

Supplementary Figures

Supplementary Table 1
