# Supplementary material for: Upregulation of HOTAIRM1 increases migration and invasion by glioblastoma cells
Source: Aging (Albany NY). 2020 Dec 11;13(2):2348–64. doi: 10.18632/aging.202263 (PMC7880397; doi:10.18632/aging.202263)
Supplement: Supplementary Figures [file aging-13-202263-s001.pdf]

SUPPLEMENTARY FIGURES

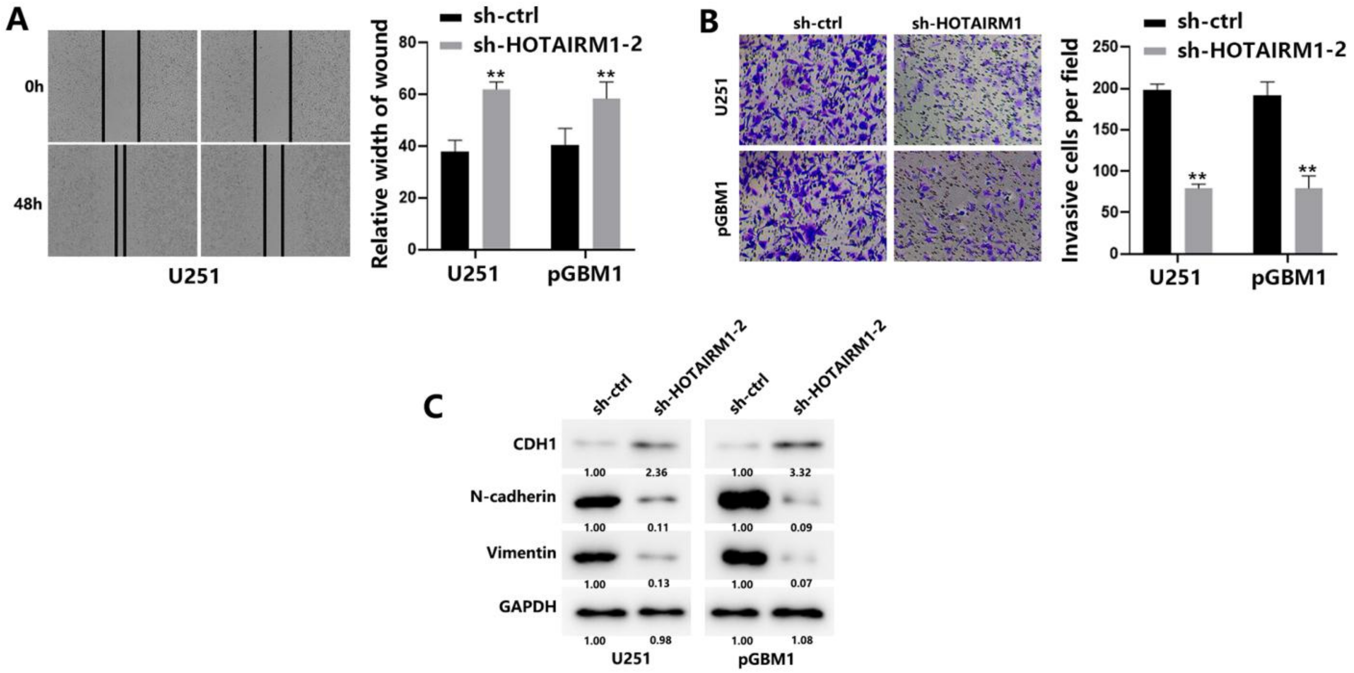

Supplementary Figure 1. (**A**) Wound healing assays were used to analyze migration of GBM cells. (**B**) Matrigel invasion assays were used to analyze invasion of GBM cells. (**C**) EMT-associated proteins in GBM cells were determined by western blotting.

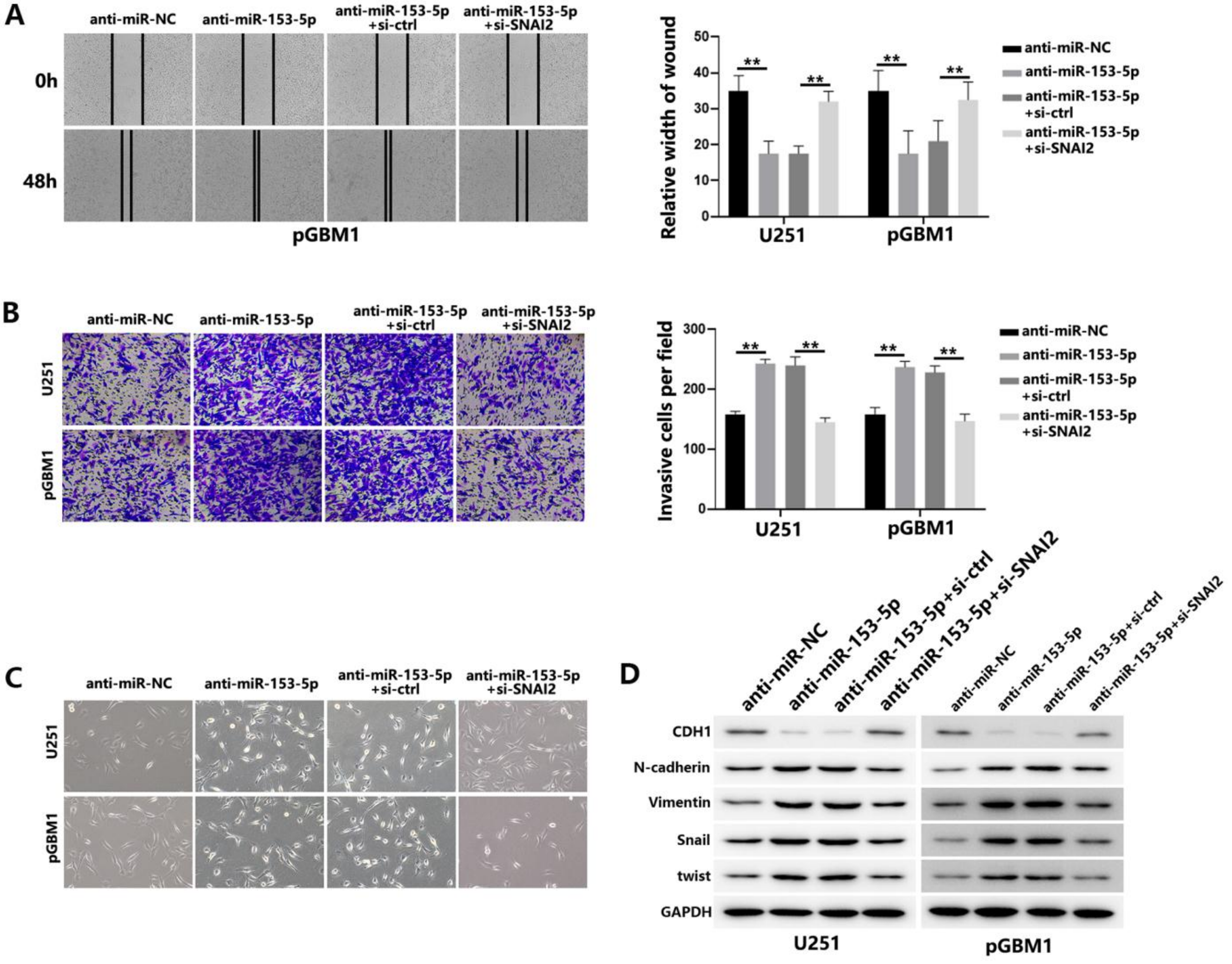

Supplementary Figure 2. (**A**) Wound healing assays were used to analyze migration of GBM cells. (**B**) Matrigel invasion assays were used to analyze invasion of GBM cells. (**C**) Morphological changes of GBM cells were imaged to analyze EMT process of GBM cells. (**D**) EMT-associated proteins in GBM cells were determined by western blotting.
