# Supplementary material for: Upregulation of HOTAIRM1 increases migration and invasion by glioblastoma cells
Source: Aging (Albany NY). 2020 Dec 11;13(2):2348–64. doi: 10.18632/aging.202263 (PMC7880397; doi:10.18632/aging.202263)
Supplement: Supplementary Table 1 [file aging-13-202263-s002.pdf]

SUPPLEMENTARY TABLE

Supplementary Table 1.

Antibodies

Name	Company	Catalog Number
CDH1	Cell Signaling Technology	#3195
N-cadherin	Cell Signaling Technology	#13116
Vimentin	Cell Signaling Technology	#5741
Snail	Abcam	ab53519
twist	Abcam	ab175430
GAPDH	Cell Signaling Technology	#5174
SNAI2	Abcam	ab85936
Ago2	Cell Signaling Technology	#2897

Primers used for qRT-PCR

Name	Forward primer	Reverse primer
HOTAIRM1	CCCACCGTTCAATGAAAG	GTTTCAAACACCCACATTTC
U6	ATTGGAACGATACAGAGAAGATT	GGAACGCTTCACGAATTTG

Primers used for CHIP

Name	Forward primer	Reverse primers
BS1	AATCTCAAGCTAACTCTTAC	CCGAATGTAGCCTTATAGAC
BS2	ACGCCTGTCCTAGGATTACT	GTAGCCTTAAGCTCGAACTT
BS3	GTTAACCGTACTAATTGGCC	CTCCCCAAATGGACTAGTCA

BS sequence

BS1: CCCACCGTTCAATGAAAG

BS2: GCGGCCGGGCTCTT

BS3: ACTCCAACCATGTGT
